# Pure high-grade papillary urothelial bladder cancer: a luminal-like subgroup with potential for targeted therapy

**DOI:** 10.1007/s13402-020-00524-6

**Published:** 2020-05-22

**Authors:** Tician Schnitzler, Nadina Ortiz-Brüchle, Ursula Schneider, Isabella Lurje, Karolina Guricova, Alexander Buchner, Gerald Bastian Schulz, Axel Heidenreich, Nadine Therese Gaisa, Ruth Knüchel, Stefan Garczyk

**Affiliations:** 1 Institute of Pathology, University Hospital RWTH Aachen, Pauwelsstr. 30, 52074 Aachen, Germany; 2 Department of Urology, Ludwig-Maximilians-University Munich, Munich, Germany; 3 Department of Urology, University Hospital Cologne, Cologne, Germany; 4 Department of Urology, University Hospital RWTH Aachen, Aachen, Germany

**Keywords:** Papillary bladder cancer, Non-muscle-invasive, high-grade bladder cancer, pTa HG, Targeted therapy, Molecular subtype, Luminal and basal markers

## Abstract

**Purpose:**

Non-invasive high-grade (HG) bladder cancer is a heterogeneous disease that is characterized insufficiently. First-line Bacillus Calmette-Guérin instillation fails in a substantial amount of cases and alternative bladder-preserving treatments are limited, underlining the need to promote a further molecular understanding of non-invasive HG lesions. Here, we characterized pure HG papillary urothelial bladder cancer (pure pTa HG), a potential subgroup of non-invasive HG bladder carcinomas, with regard to molecular subtype affiliation and potential for targeted therapy.

**Methods:**

An immunohistochemistry panel comprising luminal (KRT20, ERBB2, ESR2, GATA3) and basal (KRT5/6, KRT14) markers as well as p53 and FGFR3 was used to analyze molecular subtype affiliations of 78 pure pTa HG/papillary pT1(a) HG samples. In 66 of these, *ERBB2* fluorescence *in situ* hybridization was performed. Additionally, targeted sequencing (31 genes) of 19 pTa HG cases was conducted, focusing on known therapeutic targets or those described to predict response to targeted therapies noted in registered clinical trials or that are already approved.

**Results:**

We found that pure pTa HG/papillary pT1(a) HG lesions were characterized by a luminal-like phenotype associated with frequent (58% of samples) moderate to high ERBB2 protein expression, rare FGFR3 alterations on genomic and protein levels, and a high frequency (89% of samples) of chromatin-modifying gene alterations. Of note, 95% of pTa HG/papillary pT1 HG cases harbored at least one potential druggable genomic alteration.

**Conclusions:**

Our data should help guiding the selection of targeted therapies for investigation in future clinical trials and, additionally, may provide a basis for prospective mechanistic studies of pTa HG pathogenesis.

**Electronic supplementary material:**

The online version of this article (10.1007/s13402-020-00524-6) contains supplementary material, which is available to authorized users.

## Introduction

Bladder cancer (BC) is the most common malignancy of the urinary tract with an estimated number of 380,000 new cases and 150,000 deaths per year worldwide [1]. In Europe and North America more than 90% of diagnosed bladder cancers are urothelial carcinomas [2]. The majority of BC patients present with non-invasive, low-grade papillary carcinomas (pTa LG), characterized by frequent *FGFR3* gene alterations and a good prognosis (5-year survival of ~ 90%). Although frequently recurring, these tumors only infrequently progress to muscle-invasive disease. In contrast, muscle-invasive bladder cancer (MIBC) has an unfavorable prognosis (5-year survival < 50%) and a high risk of progression to metastasis [2]. MIBC mainly arises via carcinoma *in situ* (CIS), a flat high-grade (HG) lesion characterized by *TP53* mutations, and papillary lesions of HG (pTa HG) [2, 3]. The conventional model postulates development of pTa HG from pTa LG lesions [2]. Based on our observations and those made by others [4], we hypothesize that a subgroup of non-invasive pTa HG lesions arises *de novo* (i.e., without a LG history). The AUA/SUO (American Urological Association/Society of Urologic Oncology) and the EAU (European Association of Urology) guidelines for non-MIBC (NMIBC) strongly recommend an essentially uniform therapy for HG NMIBC: Intravesical instillation of BCG (Bacillus Calmette-Guérin) post transurethral resection of the bladder tumor (TURBT) or after confirmative re-TURBT, respectively, is the recommended first-line treatment, whereas radical cystectomy (RC) should be considered in case of BCG failure or in first-line for the subgroup of highest risk tumors [5, 6]. BCG failure is observed in a significant portion of patients [7] and even though early RC is associated with an excellent tumor-specific survival [8], not all patients are eligible for this aggressive treatment or refuse RC [9]. In sum, there is an unmet need for effective bladder-preserving alternative therapies in addition to BCG for high-risk NMIBC.

In this study and related work [10, 11], we aimed to further understand the heterogeneous group of non-invasive urothelial HG lesions as they are understudied compared to MIBC [12]. In the present study, a cohort of pure pTa HG/papillary pT1(a) HG tumors was established and analyzed with regard to molecular subtype affiliation using an established panel of luminal (KRT20, GATA3, ERBB2, ESR2) and basal (KRT5/6, KRT14) immunohistochemical markers recently used to characterize urothelial CIS [10]. Additionally, a targeted sequencing panel (recently described [11]) was applied on the pTa HG lesions, focusing on genetic alterations (GAs) in genes either encoding known therapeutic targets or genes described to predict response to targeted therapies noted in registered clinical trials or already FDA/EMA-approved. Combined with data obtained by analogous investigations on urothelial CIS, this study aims to further dissect differences and similarities within the heterogeneous group of non-invasive urothelial HG lesions that might be of relevance for therapy stratification.

## Materials and methods

### Patients

It is accepted that HG papillary carcinomas may arise from LG papillary lesions [2]. Based on our own observations and those made by other groups [4] we hypothesize that a subgroup of HG papillary lesions might develop “*de novo*” without a preceding LG component. We carefully selected a cohort of patients presenting with HG papillary urothelial BC without a history of previous or concomitant pTa LG or MIBC lesions. In two cases, a LG lesion was simultaneously present at a localization in the bladder distinctly different from the pTa HG tumor. Our retrospective immunohistochemistry (IHC) cohort comprised 78 samples from 48 patients (44 pTa HG, 34 papillary pT1(a) HG samples) treated at the RWTH Aachen University and the LMU Munich University Hospitals between 2012 and 2018 (Online Resources 1 and 2). To analyze the stability of IHC marker expression in the course of progression, we compared marker expression in matched pairs of exophytic papillary HG cancer to concomitant stroma-invasive tumor areas in 32 biopsies from 20 patients (Online Resource 1). To study the presence of GAs with potential impact on targeted therapy response, targeted sequencing was conducted in 23 samples from 23 patients, comprising 16 pTa HG/papillary pT1 HG tumor specimens without a history of pTa LG/MIBC lesions, three papillary pT1 HG samples with a history of pTa LG or muscle-invasive disease (Online Resource 3) and four normal smooth muscle specimens as controls. An experienced pathologist (RK) confirmed the diagnosis of each patient by reviewing corresponding histological samples.

### Immunohistochemistry

We used formalin-fixed, paraffin-embedded (FFPE) tissues to create tissue microarrays (TMAs). These TMAs were generated using one to five punches per sample if available to consider potential tumor heterogeneity. Negative and positive staining controls were included on all TMAs. TMA sections (2 µm) were incubated in antigen retrieval solution PT Link (Dako, Agilent, Santa Clara, California) of pH 6 (KRT14, KRT20, GATA3, ERBB2, FGFR3) and pH 9 (KRT5/6, ESR2, p53) at 95 °C for deparaffinization, rehydration and epitope retrieval. Next, slides were transferred to an automated immunostainer (Dako, Agilent) and covered with EnVision™ Flex Peroxidase Blocking-Reagent (Dako, Agilent) for five minutes to block endogenous peroxidase activity. Immunostaining was performed with antibodies specific for KRT20, GATA3, ESR2, ERBB2, KRT5/6, KRT14, p53 and FGFR3. Subsequently, tissue sections were treated with a secondary reagent (Dako, Agilent) for 15 min, followed by incubation with a horseradish peroxidase-conjugated polymer (Dako, Agilent) for 20 min. Visualization of staining was accomplished using a DAB + Substrate Chromogen System (Dako, Agilent). Finally, the TMAs were counterstained using Mayer’s hematoxylin.

Immunohistochemical staining was assessed by an experienced pathologist (RK) blinded to patient identity. The percentage of positively stained cells was evaluated for the cytoplasmic markers KRT20, KRT5/6, KRT14 and nuclear p53. Cases were considered positive for cytokeratin expression if > 50% of cells showed positivity for the respective marker. Aberrant p53 expression was assumed if either 100% of cells showed intense nuclear staining or in case of complete absence of nuclear staining (null phenotype) [13]. ESR2 and GATA3 status was assessed using an adapted semi-quantitative immunoreactive scoring system described by Remmele and Stegner [14], multiplying a score for nuclear staining intensity (from 0 to 3) with a score expressing the percentage of stained cells (from 0 to 4). A Remmele score ranging from 3 to 12 was considered “positive” as described recently [10]. The Dako score was used to quantify ERBB2 protein expression, combining staining intensity and the percentage of positive cells: 0–1 (negative), 2 (moderate) and 3 (positive, overexpressed) [15]. FGFR3 protein expression was assessed using a semi-quantitative score (0–3) recently described [16].

### Fluorescence *in situ* hybridization (FISH)

FISH analysis was applied in order to correlate ERBB2 protein expression with the genomic *ERBB2* gene status using a Zyto*Light* SPEC *ERBB2/CEN17* Dual Color Probe kit (Zytovision, Bremerhaven, Germany) on 66 pure pTa HG/papillary pT1(a) HG samples with available ERBB2 protein expression data (Online Resource 2). Concerning the stroma-invasive specimens, only the exophytic tumor area was considered. The test is based on the use of two fluorescently labeled oligonucleotide probes, one specifically binding to a centromeric region of chromosome 17 (*CEN17*) (ZyOrange) and the other targeting a sequence within the *ERBB2* gene locus on chromosome 17 (ZyGreen). The assay was conducted in accordance with the manufacturer’s instructions: FFPE tissue sections (2 µm) were dried (60 °C, overnight), followed by deparaffinization, rehydration and incubation in pretreatment buffer (98 °C for 20 min). Next, pepsin digestion (37 °C for 10 min) was performed preceding the application of *ERBB2/CEN17*-specific probes. Subsequently, complementary target DNA and FISH probes were co-denatured at 75 °C and allowed to hybridize at 37 °C overnight. After stringent washing and DAPI counterstaining, hybridized probes were visualized by fluorescence microscopy. Thirty tumor nuclei were assessed per case and a mean ratio of green (*ERBB2*) over red (*CEN17*) hybridization signals was calculated. Tumor samples were classified as “amplified” (ratio ≥ 2.2) and polysomic (polysomy 17; > 3 *CEN17* signals per nucleus) according to commonly applied thresholds [17].

### Library preparation and targeted sequencing

In order to analyze the presence of therapeutically relevant genetic alterations in a cohort of 16 pure pTa HG/papillary pT1 HG tumor specimens and three papillary pT1 HG samples with a history of LG or muscle-invasive disease from altogether 19 patients (Online Resource 3), targeted sequencing was conducted. Four normal smooth muscle specimens from four individuals without a known cancer disease served as control samples. For NGS analysis only the exophytic tumor areas were considered. The target gene panel (recently described [11]) comprised genes frequently affected in MIBC [18] and was primarily focussed on potentially druggable alterations in genes described to predict response to targeted therapies that are FDA/EMA-approved or that are in registered clinical trials (Online Resource 4). Deparaffinized tissue sections (10 µm) were prepared and target tissue material was carefully dissected. Following proteinase K (Promega, Madison, Wisconsin) digestion of isolated cell material (37 °C, overnight), DNA isolation was carried out with a Maxwell 16 instrument (Promega) using the Maxwell 16 FFPE tissue LEV DNA purification Kit (Promega). DNA was subsequently quantified using a Qubit fluorometric assay (Thermo Fisher Scientific, Waltham, Massachusetts). The quality of the isolated DNA was assessed using a TruSeq FFPE DNA Library Prep QC kit (Illumina, San Diego, California). Library preparation was performed using a TruSeq Costum Amplicon Low Input kit (Ilumina) according to the manufacturer’s instructions. In brief, 60–100 ng DNA was used for hybridization to the custom oligonucleotide pools. In order to improve sequence data validity, a dual strand sequencing protocol enabling analysis of both complementary DNA strands was applied. Normalized libraries were sequenced on a NextSeq 500 platform (Illumina) using a NextSeq 500/550 Mid Output kit (2 × 150 cycles) (Illumina). SNV analysis was performed using Sequence Pilot Software version 4.4 (SeqNext module, JSI medical systems, Ettenheim, Germany). For SNV analysis over 97% of bases had a coverage of at least 300x in the analyzed regions of interest for all samples. Missense and silent variants with an allele frequency > 2% in the normal population (according to “1000 genomes” (http://www.internationalgenome.org/; Nov 05, 2019) or “dbSNP v153” (https://www.ncbi.nlm.nih.gov/snp; Nov 05, 2019)) as well as non-splicing-relevant silent and intronic variants in general were considered “non-pathogenic”. Additionally, missense and UTR variants annotated as “benign” or “likely benign” in ClinVar (https://www.ncbi.nlm.nih.gov/clinvar/; Nov 05, 2019) were also excluded. The *TERT* gene promoter was checked for two known recurrent mutations c.-124C > T and c.-146C > T. The variant allele frequency cut-off was set to 5%. As reference genome for panel design hg19 was used, whereas hg38 served as reference genome for annotation of mutations. A recently developed in-house algorithm, based on an exponential growth model for amplification of PCR products, was applied for CNV analysis. For every gene analyzed for CNVs, at least 10 amplicons were included in the panel design. Coverage data were normalized and amplicon clustering, based on the PCR efficiency among all samples, was performed (for each amplicon). Genes were considered exhibiting a CNV if ≥ 30% of the amplicons of a gene were recognized as outliers in the majority of the five response models built within a cluster. The underlying reference genome for the panel design was hg19. The raw sequencing data of this study are deposited in the NCBI Sequence Read Archive (SRA) version 2.15.0 (https://www.ncbi.nlm.nih.gov/sra; Nov 05, 2019): accession no. PRJNA591851.

### Statistical analysis

Differential expression of luminal and basal protein markers in matched pairs of exophytic and stroma-invasive tumor areas of pure papillary pT1(a) HG carcinomas was assessed using the Wilcoxon matched-pairs signed rank test in GraphPad Prism 7.0 (GraphPad Software, San Diego, California). In order to correlate *ERBB2* genomic FISH and ERBB2 protein expression data, as well as cytokeratin expression, a two-tailed Fisher’s exact test was conducted applying SPSS Statistics software version 20.0 (IBM, Armonk, New York). For all analyses the level of significance was set to *p* < 0.05.

## Results

### Phenotypic characterization of papillary high-grade bladder cancer

**Fig. 1:**
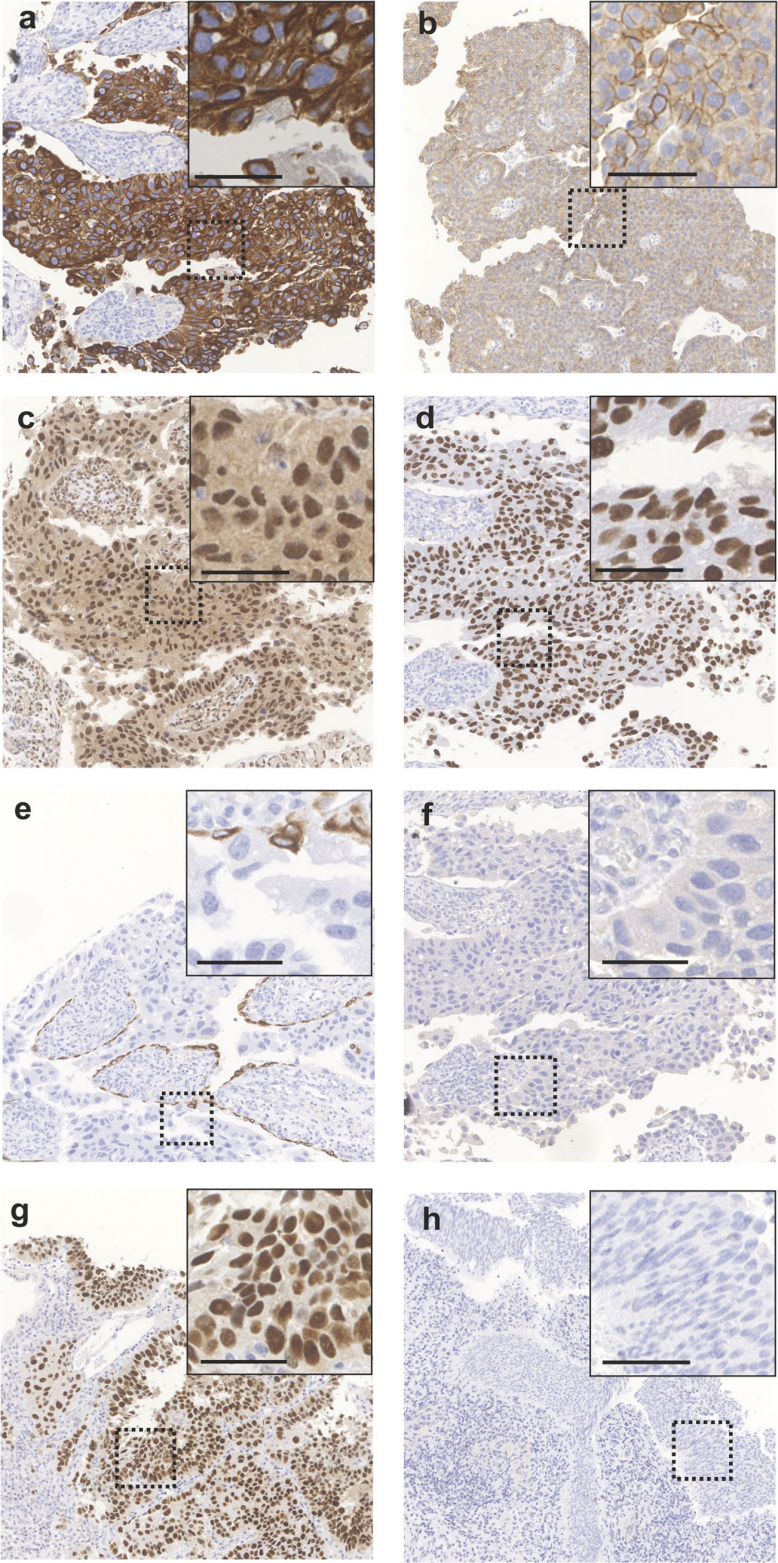
Immunohistochemical detection of luminal and basal marker expression in pure pTa HG and papillary pT1(a) HG specimens. Photographs are representative of median protein expression observed (except for p53). Aberrant KRT20 expression (**a**). ERBB2 Dako score 2 (**b**). ESR2 Remmele score 12 (**c**). GATA3 Remmele score 12 (**d**). KRT5/6 negativity in tumor cells, positivity limited to basal urothelial cells (**e**). KRT14 negative tumor cells (**f**). Aberrant p53 expression (35% of samples; null phenotype not shown) (**g**). FGFR3 negativity (**h**). Scale bar: 50 µm

In order to analyze the molecular subtype affiliation of pure pTa HG/papillary pT1(a) HG tumors, extensively studied for MIBC recently [18, 19], protein expression of luminal (KRT20, ERBB2, ESR2, GATA3) and basal markers (KRT5/6, KRT14), as well as of p53 and FGFR3, was examined in a cohort of 78 samples (44 pTa HG and 34 papillary pT1(a) HG samples) from 48 patients (Online Resources 1 and 2). For stroma-invasive papillary HG samples, only expression in exophytic tumor areas was considered. The results are summarized in Table 1 and compared to marker expression recently described for urothelial CIS [10]. Representative images for IHC marker expression in pure pTa HG specimens and exophytic tumor areas of pure papillary pT1(a) HG samples, respectively, are shown in Fig. 1. We found that the majority of samples exhibited positivity for luminal markers: in 74% (58/78) of the samples an aberrant positivity for KRT20 was observed and 99% (77/78) of the samples were scored as “positive” for GATA3 expression. Additionally, positive staining for ESR2 was noted in 96% (75/78) and ERBB2 overexpression (Dako score 3) was observed in 28% (22/78) of the samples. In contrast, the majority of specimens lacked expression of basal markers: 3% (2/78) and 9% (7/78) stained positive for the basal cytokeratins KRT5/6 and KRT14, respectively. A correlation of luminal and basal cytokeratin expression is shown in Online Resource 5. Aberrant p53 expression was detected in 35% (27/78), whereas high FGFR3 protein expression was observed in 10% (7/72) of the samples. Taken together, we found that pure pTa HG/papillary pT1(a) HG carcinomas are characterized by a luminal-like IHC-based phenotype, comparable to that observed for urothelial CIS.

**Table 1:** Expression of luminal and basal protein markers in pure pTa HG and pure papillary pT1(a) HG in comparison to urothelial CIS samples [10]

	pTa HG/papillary pT1(a) HG samples		median	CIS samples		median
	n = 78^a^	(100%)		n = 156	(100%)	
KRT20						
negative	20	(26%)	positive	24	(15%)	positive
positive	58	(74%)		132	(85%)	
ERBB2						
0–1	33	(42%)	2	55	(35%)	2
2	23	(30%)		51	(33%)	
3	22	(28%)		50	(32%)	
ESR2						
0–2	3	(4%)	12	18	(12%)	12
3–12	75	(96%)		138	(88%)	
GATA3						
0–2	1	(1%)	12	5	(3%)	12
3–12	77	(99%)		151	(97%)	
KRT5/6						
negative	76	(97%)	negative	153	(98%)	negative
positive	2	(3%)		3	(2%)	
KRT14						
negative	71	(91%)	negative	154	(99%)	negative
positive	7	(9%)		2	(1%)	
p53						
non-aberrant	51	(65%)	-	57	(37%)	-
aberrant	27	(35%)		99	(63%)	
FGFR3				-	-	-
negative/low	65	(90%)	negative			
high	7	(10%)				

Overview of protein expression of luminal and basal markers as well as of p53 and FGFR3 in a cohort of pure pTa HG and pure papillary pT1(a) HG samples. Only marker expression in exophytic tumor areas was scored. Data are compared to marker expression in a recently characterized CIS cohort [10]. a: For FGFR3, protein expression in 72 samples was analyzed

Recently, a potential switch from luminal to basal marker expression during stromal invasion of urothelial CIS was noted [10]. When comparing the expression of the above mentioned marker proteins (except for p53) in matched pairs (n = 32 biopsies from 20 patients) of exophytic and concomitant stroma-invasive tumor areas (Online Resource 1), no significant difference in the expression of marker proteins, except for a reduction in KRT20 protein expression in the stroma-invasive tumor area (*p* < 0.01), was noted (Online Resource 6).

### *ERBB2* fluorescence *in situ* hybridization of papillary high-grade tumors

**Fig. 2:**
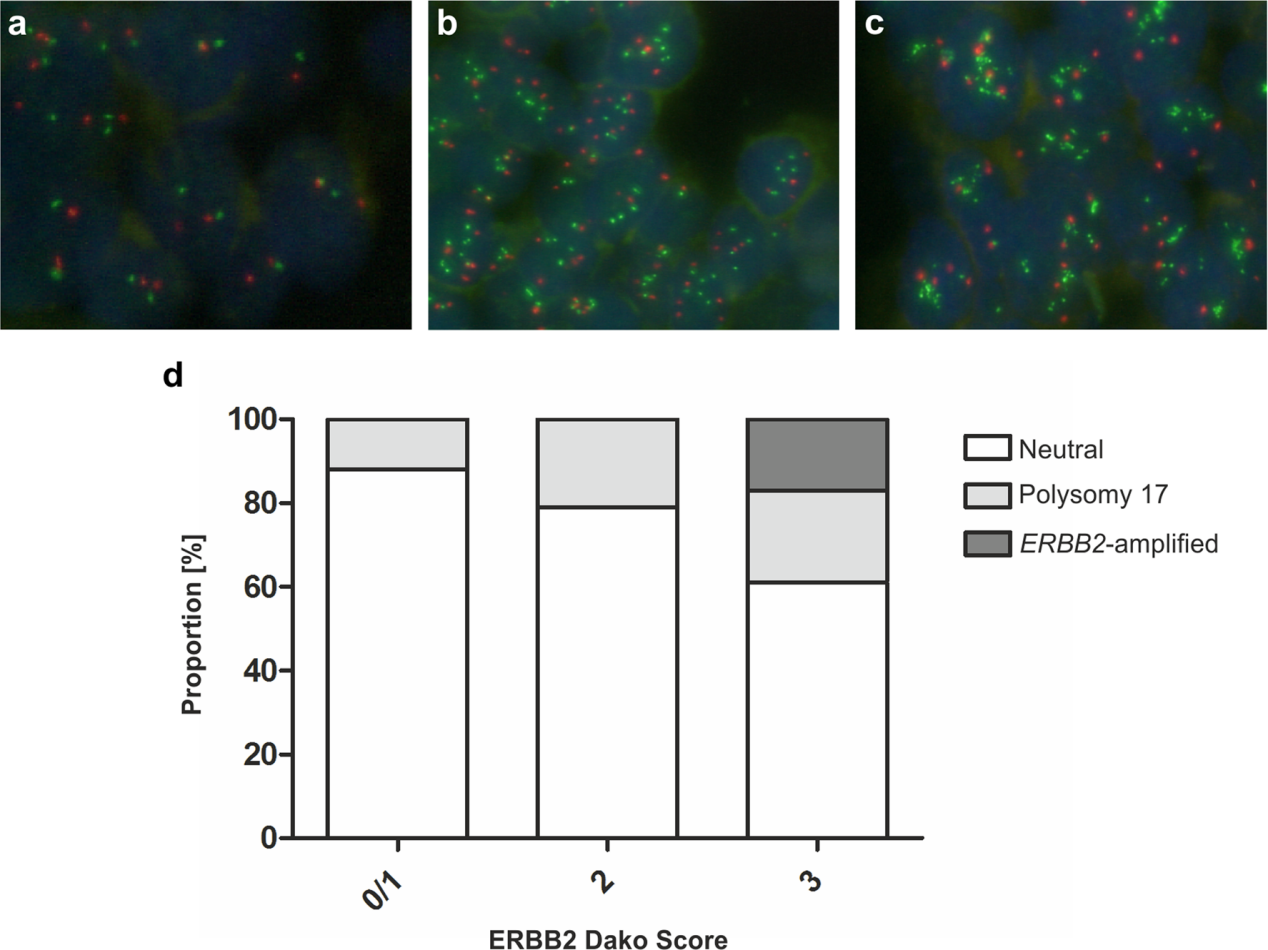
*ERBB2* fluorescence *in situ* hybridization analysis (n = 66 samples). Neutral (non-amplified, non-polysomic for chromosome 17) (**a**), polysomy 17 (**b**), *ERBB2* amplification (**c**), distribution of *ERBB2*-amplified, chromosome 17 polysomic and neutral samples among the ERBB2 Dako score groups “0/1”, “2” and “3” (**d**). Red hybridization signals indicate the centromeric region of chromosome 17 (*CEN17*), green signals localize to the *ERBB2* gene locus on chromosome 17

To correlate protein expression levels of the known therapeutic target ERBB2 with potential underlying *ERBB2* locus amplification or polysomy 17, *ERBB2* fluorescence *in situ* hybridization was performed in 66 pure pTa HG and pure papillary pT1(a) HG samples (Online Resource 2). Concerning stroma-invasive specimens, only the exophytic tumor part was considered. A polysomy 17 was observed in 17% (11/66) and *ERBB2* gene locus amplification in 5% (3/66) of the specimens. Although no statistically significant correlation between ERBB2 protein expression (Dako score 0,1 + vs. 2+, 3+) and the presence of *ERBB2* amplification or polysomy 17 (neutral vs. AMP/Poly 17) was observed (two-tailed Fisher’s exact test: *p* = 0.073), we noted that all *ERBB2*-amplified specimens showed ERBB2 protein overexpression (Dako 3+) (Table 2; Fig. 2).

**Table 2:** ERBB2 protein expression in 66 pure pTa HG and pure papillary pT1(a) HG tumors analyzed by *ERBB2* FISH

Dako score		0,1+	2+	3+
Neutral	n = 52	26	15	11
Polysomy 17	n = 11	3	4	4
*ERBB2*-amplified	n = 03	0	0	3

ERBB2 protein expression was quantified using the Dako score (0–3+). Samples were categorized into “neutral”, chromosome 17 polysomic (“polysomy 17”) and “*ERBB2*-amplified” tumors using fluorescence *in situ* hybridization (FISH) for *ERBB2* and *CEN17*. Concerning the stroma-invasive samples, only the exophytic tumor area was considered for analysis

### Potentially actionable genomic alterations in papillary high-grade bladder cancer

Next, we applied a targeted sequencing panel that was recently used to characterize urothelial CIS [11], focusing on genetic alterations (GAs) in genes either encoding known therapeutic targets or that are described to predict response to targeted therapies (currently in registered clinical trials or FDA/EMA-approved). The tissue cohort comprised 19 FFPE tumor biopsies, each sample taken from an individual bladder cancer patient (16 pure pTa HG/papillary pT1 HG tumor specimens and three samples from cases with a history of LG or MIBC disease), and four normal smooth muscle control specimens from patients without a history of cancer (Online Resource 3). For targeted NGS analysis only the exophytic tumor areas were used.

**Fig. 3:**
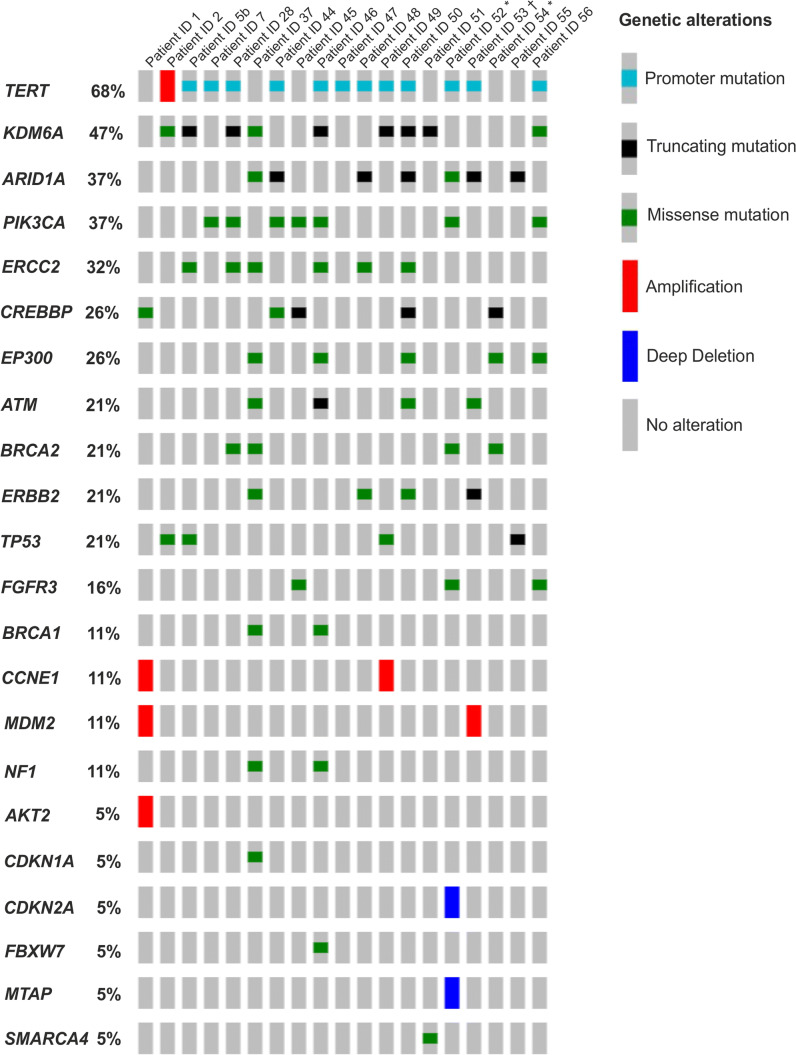
Mutations and CNVs detected in a cohort of 19 pTa/papillary pT1 HG samples. *: Patient with pTa LG history; †: Patient with history of MIBC

Analysis of mutations and CNVs in 31 genes (Online Resource 4) revealed 96 genetic alterations in total, including multiple aberrations per gene in a given sample. On average, pTa HG/papillary pT1 HG samples harbored 5.1 alterations (range: 1–13) (Online Resources 7 and 8). Affected genes per patient are summarized in Fig. 3. *TERT* gene promoter mutations, detected in 63% (12/19) of the cases, were identified to be the most frequent GAs in our analysis (Fig. 3). Moreover, in 5% (1/19) of the cases *TERT* gene amplification was observed. Genes encoding chromatin-modifying proteins (*KDM6A*, *ARID1A*, *CREBBP*, *EP300*, *SMARCA4*) were impacted with a frequency of 89% (17/19) of the cases, followed by 63% (12/19) of specimens harboring at least one mutation in genes of the PI3K/MAPK pathway (*PIK3CA*, *ERBB2*, *FGFR3*, *NF1, AKT2*). Moreover, DNA damage response (DDR) genes (*ATM*, *ERCC2*, *BRCA2*, *BRCA1*) and genes of the *TP53*/cell cycle pathway (*TP53, CCNE1, MDM2, CDKN1A, CDKN2A, FBXW7*) exhibited variants in 47% (9/19) of pTa HG/papillary pT1 HG specimens, respectively.

**Fig. 4:**
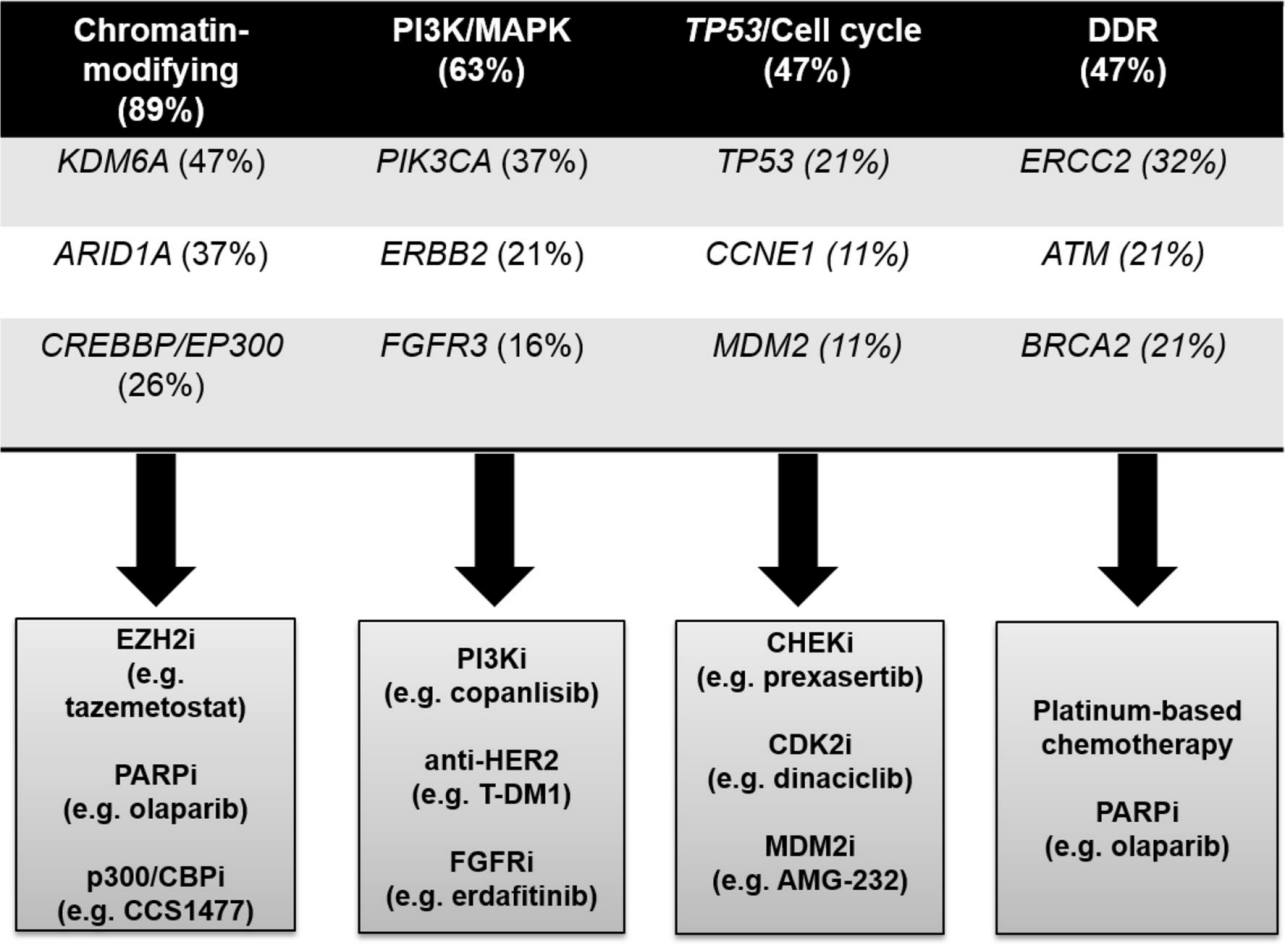
Potentially actionable mutations detected in pTa HG/papillary pT1 HG samples and suggested targeted therapy. Impacted pathways and frequency of cases exhibiting at least one potentially actionable alteration in the respective pathway (upper part). The three most frequently affected genes of each pathway are depicted. Potential targeted therapies are shown (lower part)

86% (83/96) of all alterations were considered “potentially actionable” using targeted therapies, including variants of yet unknown significance in genes with predictive potential (Online Resources 7 and 8). On average, 4.4 potentially actionable alterations were detected per patient (range: 0–13) and 95% (18/19) of pTa HG/papillary pT1 HG cases exhibited at least one alteration in genes potentially impacting the selection of targeted therapies. Frequently impacted genes found in pTa HG/papillary pT1 HG cases that were considered “potentially actionable” and related potential targeted therapies are summarized in Fig. 4.

## Discussion

Non-invasive urothelial HG bladder cancer constitutes a heterogeneous group of tumors (i.e., flat urothelial CIS and pTa HG lesions [2]) that is characterized insufficiently [12]. Bladder-preserving treatment options in addition to intravesical BCG instillation are limited and reliable prognostic markers are lacking [5], underlining the need to specifically promote molecular understanding of non-invasive HG lesions. A carefully diagnosed non-invasive state is the best justification for local and bladder-preserving therapy since the chance of metastasis has not yet been taken into account. In the present study, we distinctly selected a cohort of pure pTa HG/papillary pT1(a) HG tumors, a potential subgroup of pTa HG carcinomas without a history of previous or concomitant pTa LG or MIBC disease.

First, we aimed to analyze this putative subgroup with regard to molecular bladder cancer subtype affiliation described for MIBC [18], applying an immunohistochemistry (IHC) panel recently used to characterize urothelial CIS [10]. The majority of pure pTa HG/papillary pT1(a) HG samples exhibited a luminal-like phenotype, i.e., positivity for KRT20 and a lack of basal cytokeratin (KRT5/6 and KRT14) expression in tumor cells. This phenotype is comparable to that observed for urothelial CIS [10] and in accordance with recent studies in NMI urothelial papillary carcinomas of the urinary bladder [20] and the upper tract [21], describing an association between a luminal-like phenotype and high tumor cell grade. Importantly, a luminal-like phenotype has been correlated with unfavorable patient outcome in NMIBC [22–24] and NMI urothelial papillary cancer of the upper tract [21, 25], in contrast to observations made in MIBC [18]. As no follow-up data were available for the here analyzed, carefully selected patient cohort, it will be interesting to see in upcoming studies if luminal-like pure pTa HG tumors are indeed associated with a worse prognosis compared to non-luminal cases within this subgroup.

The here studied pTa HG/papillary pT1 HG samples and unselected pTa HG specimens of a recently published reference cohort [26] exhibited a similar pattern of alteration frequencies in the analyzed genes. Remarkably, pTa HG/papillary pT1 HG tumors in the present study and the reference cohort showed a high frequency of alterations in genes encoding chromatin-modifying proteins, including *KDM6A*, *ARID1A*, *EP300* and *CREBBP*, which is in line with a recent report analyzing a mixed cohort of urothelial papillary bladder cancers of low and high grade [27]. Notably, pTa HG/papillary pT1 HG samples in this study showed a lower alteration rate in the *FGFR3* (16% vs. 59%) and *CDKN2A* (5% vs. 22%) genes compared to unselected pTa HG lesions [26]. In accordance with this observation, a low rate (7/72; 10%) of high FGFR3 protein expression was noted in the present study. Alterations in *FGFR3* and *CDKN2A* have been shown to co-occur [28] and to be implicated in the conventional Ta developmental pathway, whereby Ta HG tumors evolve from precedent LG lesions [2]. Of note, a recent study suggested *CDKN2A* deletions to appear prior to grade progression in Ta LG lesions [29]. The observed reduction in *FGFR3* mutational frequency in unselected pTa HG lesions (59%) compared to pTa LG lesions (~ 70–80%) [26, 30] may be explained by a putative higher rate of grade progression in LG cell clones without *FGFR3* alterations compared to those harboring *FGFR3* mutations and/or a putative separate developmental pathway of Ta HG lesions. Interestingly, a recent study found that the same *FGFR3* mutations were detectable in LG and adjacent HG areas of the same tumors [29], suggesting grade progression of *FGFR3*-mutated cell clones. Of note, one of the three here analyzed *FGFR3*-mutated samples (patient ID 52, harboring the only *CDKN2A* deletion in the cohort) was known to have a LG history. In future studies it will be interesting to corroborate these data by analyzing *FGFR3* mutational frequencies in larger cohorts of pure pTa HG without LG and MIBC history and also by including papillary bladder cancers with grade heterogeneity.

Papillary HG tumors and CIS are concurrently diagnosed in a substantial portion of patients [26]. In this and a preceding study in urothelial CIS [11], we aimed to identify GAs having the potential to guide investigation of targeted therapies in future clinical trials in high-risk NMIBC. In contrast to CIS, the here analyzed pTa HG/papillary pT1 HG samples were characterized by a higher mutational rate in PI3K/MAPK pathway genes (63% vs. 36%), mainly due to a higher mutational frequency in the *PIK3CA* and *FGFR3* genes [11]. Of note, *FGFR1* amplifications were exclusively found in CIS specimens [11]. Moreover, CIS samples exhibited a higher mutational rate in genes of the *TP53*/cell cycle pathway compared to pTa HG/papillary pT1 HG lesions (72% vs. 47%), mainly due to differences in the alteration frequencies of *TP53* and *CCND1* [11]. These observations are in accordance with the accepted model of a distinct development of papillary bladder cancer and CIS [2]. Both, pTa HG/papillary pT1 HG tumors in this study and CIS specimens were characterized by a high proportion of alterations in chromatin-modifying genes (89% and 68%), with a significantly higher percentage of *KDM6A* alterations in pTa HG/papillary pT1 HG tumors [11]. Additionally, both lesions exhibited frequent alterations in DDR genes, with CIS lesions showing a higher mutational rate in homologous recombination (HR)-related genes (*BRCA1*, *BRCA2*, *ATM*), whereas pTa HG/papillary pT1 HG lesions specifically exhibited a high frequency of *ERCC2* mutations [11].

Of note, 95% of the analyzed pTa HG/papillary pT1 HG samples in this study exhibited at least one GA potentially predicting response to targeted therapy. Shared (in pTa HG/papillary pT1 HG and CIS) frequent alterations in the chromatin-modifying genes *KDM6A*, *ARID1A*, *CREBBP* and *EP300* should encourage clinical testing of inhibitors of EZH2 [31], PARP [32] and EP300/CREBBP bromodomain [33] for high-risk NMIBC, having either entered clinical trials (https://clinicaltrials.gov/; Oct 25, 2019; NCT03854474, NCT03568656) or being (in the case of PARP inhibitors) FDA-approved. Likewise, the high percentage of alterations in the HR-related *BRCA2*, *ATM* and *BRCA1* genes in both HG entities [11] underlines the potential of PARP inhibitors [34]. As suggested recently for urothelial CIS [10, 11], anti-ERBB2 therapy, especially the use of antibody-drug conjugates (e.g. T-DM1), might be a rational therapy for high-risk NMIBC. A substantial portion of both lesions exhibited moderate to strong positivity for ERBB2 protein (58% and 65% of samples), mutations including known pathogenic variants as well as polysomy 17 and, less frequently, *ERBB2* amplifications. Although *TP53* mutations are more frequent in CIS [2], a significant portion (~ 20%) of pTa HG/papillary pT1 HG tumors in this study and a recent work [26] harbored alterations in this gene. As suggested for urothelial CIS [11], *TP53*-mutated pTa HG tumors might also be vulnerable to WEE1/CHEK1 inhibition [35, 36]. Additionally, *MDM2* amplifications were detected in a similar limited fraction of CIS [11] and the here analyzed pTa HG/papillary pT1 HG samples (~ 10–15%), justifying testing of MDM2 inhibitors in clinical trials. *PIK3CA*, one of the top mutated genes (37%) found in this study, is impacted in a limited percentage (12%) of CIS specimens [11] as well. FDA-approved PI3K inhibitors are thus assumed to be highly effective in targeting pTa HG/papillary pT1 HG and, to a lesser extent, CIS cells [37]. In addition to targeted strategies potentially impacting flat and papillary HG lesions, actionable GAs being more specific for pTa HG/papillary pT1 HG lesions were also identified. These included GAs in *FGFR3*, *CCNE1*, and *NF1* that might be predictive of response to FGFR [38], CDK2 [39] and MTOR inhibitors [40]. Remarkably, a high frequency of *ERCC2* (32%) mutations was detected as noted recently [26] that have been found to be associated with an improved response to platinum chemotherapy in advanced urothelial carcinoma [41].

Larger-scale studies are needed to validate these preliminary findings, also by including papillary lesions with grade heterogeneity. NMI urothelial HG lesions frequently co-occur in the urinary bladder of the same patient and it will be important to analyze if the here identified potentially actionable GAs are detectable in concurrent NMI HG lesions of the same patients. Moreover, due to our primary motive of identifying therapy targets, matched control samples were not included for pTa HG/papillary pT1 cases and a certain discrimination of germline and somatic variants was thus not possible.

In summary, non-invasive, pure high-grade papillary urothelial bladder cancers, characterized by a luminal-like phenotype, a low *FGFR3/CDKN2A* alteration frequency and a high rate of mutations in genes encoding chromatin-modifying proteins, harbor at least one potentially actionable GA in the majority of cases. Our previous similar findings in CIS encourage the idea of new strategies for bladder-preserving therapy. The data presented should help guiding the selection of targeted therapies that should be investigated in future clinical trials.

## Electronic supplementary material

Online Resource 1 (XLSX 13 kb)

Online Resource 2 (XLSX 16 kb)

Online Resource 3 (XLSX 11 kb)

Online Resource 4 (XLSX 10 kb)

Online Resource 5 (XLSX 10 kb)

Online Resource 6 (PDF 170 kb)

Online Resource 7 (XLSX 20 kb)

Online Resource 8 (XLSX 10 kb)
