# Supplementary material for: Pure high-grade papillary urothelial bladder cancer: a luminal-like subgroup with potential for targeted therapy
Source: Cell Oncol (Dordr). 2020 May 22;43(5):807–19. doi: 10.1007/s13402-020-00524-6 (PMC7581583; doi:10.1007/s13402-020-00524-6)
Supplement: Supplementary file 6 — (PDF 170 kb) [file 13402_2020_524_MOESM6_ESM.pdf]

Cellular Oncology

Pure high-grade papillary urothelial bladder cancer: a luminal-like subgroup with potential for targeted therapy

Tician Schnitzler, Nadina Ortiz-Brüchle, Ursula Schneider, Isabella Lurje, Karolina Guricova, Alexander Buchner, Gerald Bastian Schulz, Axel Heidenreich, Nadine Therese Gaisa, Ruth Knüchel and Stefan Garczyk

Corresponding author:

Stefan Garczyk, PhD, Institute of Pathology, University Hospital RWTH Aachen, Pauwelsstr. 30, 52074 Aachen, Germany, Email: sgarczyk@ukaachen.de

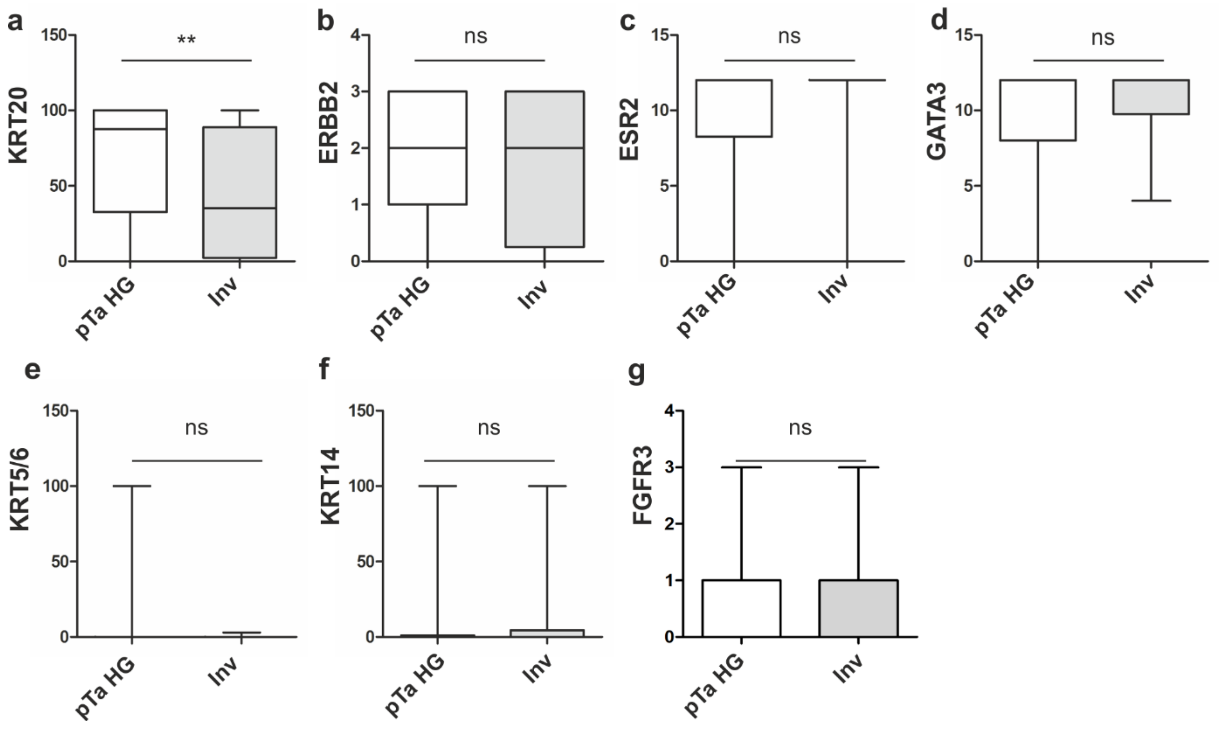

Online Resource 6 Marker expression in exophytic (pTa HG) and concomitant stroma-invasive tumor (Inv) areas of pure papillary pT1(a) HG carcinomas. Expression in n=32 matched tissue pairs was analyzed for each marker. KRT20 (percentage of positive cells) (**a**). ERBB2 (Dako score) (**b**). ESR2 (Remmele score) (**c**). GATA3

(Remmele score) (**d**). KRT5/6 (percentage of positive cells) (**e**). KRT14 (percentage of positive cells) (**f**). FGFR3 (semi-quantitative score) (**g**). Horizontal lines of boxes indicate median values, bottom and top of boxes show 25% and 75% quartiles, whiskers illustrate range of data distribution (minimum/maximum). ** $P < 0.01$, ns: not significant (Wilcoxon matched-pairs signed rank test).
